# Observations on the Structure of the Brain, Comprising an Estimate of the Claims of Drs. Gall and Spurzheim to Discovery in the Anatomy of That Organ

**Published:** 1817-07

**Authors:** 


"*Observations on the Structure of the Brain, comprising an Estimate of the Claims of Drs. Gall and Spurzheim to Discovery in the Anatomy of that Organ.* By JOHN GORDON, M. D. F. R. S. E. Lecturer on Anatomy and Surgery, and on the Institutions of Medicine; Member of the Royal College of Surgeons, of Edinburgh; and one of the Surgeons to the Royal Infirmary." 8vo. pp. 215, Edinburgh, 1817.

---

"*Examination of the Objections made in Britain against the Doctrines of Gall and Spurzheim.* By J. G. SPURZHEIM, M. D." 8vo. pp. 87. Edinburgh, 1817.

THE researches of Drs. Gall and Spurzheim in Cerebral Anatomy and Pathology, and the novelty of the opinions which they have offered to the world as the result of their labours, have excited a strong interest in the principal schools of Europe. The great importance attached to their opinions by many deservedly eminent anatomists and physicians, together with the merit of the subject in itself, considered as an advance in science, has convinced us that we can no longer with-hold a detailed explanation of their peculiar views, without being guilty towards our readers of *misprision* of information, which it much imports them to know: for, whatever noise the investigations in question have made in this or in other countries, we presume we are correct when we say that the majority of our readers, and of the profession in general, are very imperfectly acquainted with the accessions which our knowledge has lately received from these Gentlemen, on this obscure and very difficult portion of anatomy.

It is this consideration which has suggested the present article; and in our estimation it is a consideration of so weighty a nature, as to overcome the reluctance which we feel in taking up any subject that is assailed and hampered by controversy; for, be it known, that the labours of these anatomists have excited a controversy of as acrimonious a temper (on one side at least) as any that we at present remember to have occurred in recent times. But in the exercise of our office, we shall endeavour to mingle in it as little as possible. Duty indeed calls upon us for the expression of an opinion, but in that expression we shall be guided by soberness and candour. We have no inveteracy of prejudice to indulge, no resentment to gratify, no incautious positions or tottering theories to prop up, and no indirect purpose to serve. Therefore, in our examination of the subject-matter of the two Treatises under review, our only aim shall be the exposition of truth, and the furthering of science.

The names of Gall and Spurzheim have, for a series of years past, been familiar to our readers; but it is only since the latter Gentleman commenced his lectures amongst us, that the true nature and extent of their investigations have been rightly apprehended by the anatomists of this country. Their large work, published at Paris in the year 1810, had, to be sure, previously circulated to a limited extent; but when the extreme complexity of the cerebral mass is borne in mind, and the inadequacy of plates, however well executed, and of verbal descriptions, though ever so accurate, to teach what can only be seen by a peculiar mode of dissection, not heretofore in general use, and that mode performed with peculiar nicety, we can hardly wonder that their discoveries should have taken but an imperfect hold of the public mind. We know, indeed, that some who attempted, merely from their descriptions and delineations, to dissect the brain in their improved manner, failed altogether, and in consequence were led to deny the reality of the appearances professed to be demonstrable; and it was only after they had repeatedly seen Dr. Spurzheim demonstrate that organ, that they were able to trace out, to their own conviction, the structure which he so adroitly and satisfactorily exhibits.

From all this it is apparent, that if the Gentlemen in question wished to propagate their opinions, they must do something besides putting forth books or plates on the subject; and we are of opinion, that Dr. Spurzheim has acted wisely in visiting in succession the various countries and states of Europe, for the purpose of bringing, by actual and ocular demonstration, his reviews home to anatomists. The measure was indeed absolutely indispensible to the object in question; without it, his ideas could never make their way, seeing they could never be sufficiently understood. The indecent reproach of empiricism, the petty inuendoes about "locomotion," and the insinuated irrespectability of "having no fixed city," which his conduct in this respect has drawn upon him from minds of a certain order, in our opinion he has done well to treat with silent disregard. *Buchanan* and *Milton,* names to whom future ages will continue to bow, as past ages have bowed, visited the celebrated academies of their time for purposes of science; and by the public display of their mighty genius, gave the most exalted idea of their country's literature and their own. The practice indeed was formerly common with many other distinguished men, who have done honour to learning; and we are sure we shall be supported by the good sense and good feelings of our readers, when we say, that to have followed an example *so* sanctioned, and to have prosecuted science at the expence of personal interest, and personal comfort and convenience, ought never to be listened to for a moment as a matter of accusation, or ground of reproach, against any individual.

We have before observed, that the views of these eminent anatomists have been assailed by the weapons of controversy. The attack was some time since commenced in the *Reviews* of the day, and it was commenced in a tone which we are ever sorry to see introduced into scientific descriptions. It has been kept up by Dr. Gordon in the work before us, and, as may be seen from the leash of titles and decorative adjuncts that grace its first page, he is an antagonist of no small consequence. With the youthful freshness of a *volunteer* in the cause, he has gone through a power of labour, has rummaged the folios of past ages, and compelled, as it were, the older anatomists to pronounce in his favour from their graves! After pointedly and altogether denying, in "the Edinburgh Review" for June, 1815, p. 227 (for Dr. G. is well known to be the author of that notorious article), the truth or reality of the greater part of the descriptions and delineations of the brain, given by Drs. Gall and Spurzheim, he very dexterously, in the publication before us, veers round, and declares, that though their descriptions are essentially true, they were well known to *Vieussenius, Santorini, Lieutaud, Haller, Malpighi, Portal, Vicq.D'Azyr, Reiley,* and others, and that consequently all the merit is due to these anatomists, while nothing is left to Gall and Spurzheim but a bitter *residuum* of reproach for having appropriated to themselves the discoveries of their betters!—Now is all this quite so? Have we indeed sipp'd the stream of Lethe, and quite forgotten ourselves to stone? or are we such flimsy "stuff as dreams are made of?" Since these things were so well known from the writings of the older anatomists, have we not reason to wonder why they were not taught in our schools? for, to the best of our remembrance, they were not taught when we studied anatomy.

These questions we shall duly consider hereafter; meanwhile we must gratify our readers, who may not have seen Dr. Spurzheim's demonstrations of the recent brain, with a brief intelligible account of his manner of procedure, and of what he exhibits.

The controversy, however, of which we have just given the outlines, has so forcibly struck our minds, and the spirit it betrays so powerfully speaks to our associations, that before setting about our proper business, we must beg to stop a moment by the way, just to remark upon what must be considered the hard fate of the votaries of knowledge. We do not now allude to that wayward fortune, which too often smiles not on the man of genius, but consigns him to poverty and neglect during his life, while it leaves his memory to the tardy justice of posterity, to be illustrated by the cold memorial of the sculptured bust, or the empty honours of the cenotaph. We refer rather to that jealousy of opposition and malignity of rivalship, that envy and detraction, which so generally pursue merit when it has distinguished itself in the forward path of discovery or improvement. An individual so gifted is to be admired and imitated, but he cannot be envied. What suffering must he not brook! Predilections stand in his way, received opinions rise up against him, and he has to contend, with no very good hopes, against that peculiar stiffness, contortion, and preoccupation of the understanding, which Lord Bacon has, quaintly enough, called "the idols of the den" *(idola specûs)*; an idol which inspires its votaries with motives and tendencies almost independently of their own consciousness; an idol that demoralizes the feelings, and hardens the intellect against the impressions and reception of truth. Thus, authority is paramount, and prejudice triumphant; and thus the march of improvement is opposed by the prepossessions of education, and the stagnant calm of established, though possibly erroneous, opinion. A discovery is first asserted to be not true; when this can no longer be maintained, it is said to be not new! It will be remembered, that the detractors of *his* day tried all in their power to wrest the palm from the immortal *Harvey*, by giving gratuitously to *Fabricius ab Aquapendente*, and other preceding anatomists, that discovery which his genius first conceived, and his diligence perfected by zealous experiment. Those who are versed in the history of medicine will also recollect what violent opposition was made to the introduction of those valuable remedies, blisters and Peruvian bark, into the cure of diseases; and how the first employment of mercury so shocked the prejudices of the Faculty of that time, that it became a subject of virulent controversy, and many of them (sincere worshippers of the *idola specûs)* wilfully shut their eyes against the proofs of daily experience, deeming it more honourable, forsooth! to be "in the wrong with Galen, than be in the right with Paracelsus." But we need not turn over the medical annals of the days that are gone, for illustrations of this blind and baneful spirit: the youngest in the profession may remember the illiberal opposition to Dr. Jenner's immortal discovery. In a like manner, Dr. Currie's discovery of the efficacy of cold water in typhus was undervalued; and when the merit of it could no longer be denied, its first use was attempted to be traced even to Hippocrates himself. (How *far* will not this spirit go!) Dr. Jackson, again, was traduced and ridiculed for having introduced, or extended, in the army, the practice of venæsection in fevers, though the utility of that practice is now as apparent as the light of day. At a still more recent period, has Mr. Doughty (see *Medico-Chirurg. Journal*, vol. iii.) been persecuted for his opinions, and for his conduct to patients whom he cured: and at a period equally recent, has Dr. Spurzheim drawn down upon himself the ire of the Reviewer, and the mock-thunder of the Lecturer on Anatomy ("tantæne animis cœlestibus iræ?) for daring, in this enlightened age of philosophy, when, of course, we know every thing, to bring forward new conclusions with regard to the structure and functions of the brain. Moreover, for his unpardonable boldness in calling in Nature as an arbiter, and demonstrating the brain under the noses of his opponents (an offence that, 'twould seem, "*smelt* rank to Heaven"), he has been visited by a *trimming* octavo, which, though slender in size, is really stored with very *weighty* matter.

Upon the whole, then, the history of past ages, as well as of the present, shews, that opposition made to any discovery or improvement in science is, in general, no test of its demerit, but rather the contrary. Ainsi va le monde: so it has always been, and thus it ever will be.

It is high time, however, that we should turn from such reflections, which tend to give but dark and uncomfortable prospects of the literary lot, to the subject proposed, namely, a succinct descriptive outline of Dr. Spurzheim's demonstration of the brain and spinal marrow.

It is one of his fundamental positions, that the brain is a double organ, and furnished with a double apparatus of instruments. According to this view, each hemisphere corresponds, in point of structure, with its fellow of the opposite side, and has a similarity of function assigned to it. They are in communication with each other, through the medium of the commissures.

It is another fundamental position with him, that every part of the nervous system exists for itself, independently of any other part: hence no one nerve can be said to *take its origin* from another, as if it *grew* therefrom; but merely to be in *communication*. Anatomical books have hitherto spoken improperly of the origin of the cerebral and spinal nerves, as if these were merely prolongations of the brain and medulla spinalis. But the numerous instances of acephalous fœtuses, in which the thoracic and abdominal nerves are found complete; and the anatomy of some of the lower animals (the oyster and some insects for instance), which have a nervous system, though they have neither brain nor spinal marrow; completely disprove such an idea. Indeed, in the present state of our knowledge, it would be absurd to deny the independent existence of every portion of the nervous system, brains spinal marrow, or nerves in general; because no one part exists before or after other, but all have their separate and progressive developement, according to their respective functions, and are merely brought into communication with each other. In this respect, therefore, the opinion of Drs. Gall and Spurzheim is consentaneous to sound philosophy and extended observation.

The nerves of each side of the body are united to each other by means of the plexuses in the great cavities, and of the commissures in the brain; but the great centre of communication of all the nerves in the body, superior and inferior, Dr. Spurzheim maintains, is the medulla oblongata, and upper portion of the spinal marrow. In this spot, he shews, is the true origin (to use the common language) of the nerves hitherto considered cerebral, and not where they penetrate the surface of the brain-proper; and from this spot emanate those nervous fasciculi, which, by repeated accessions of fibres in their progress upwards, are ultimately expanded, to constitute the mass of the hemispheres of the brain and cerebellum.

According to this leading idea, therefore, he begins his demonstration at the medulla oblongata. By cutting the investing membranes, and gently separating its anterior fissure, he points out the *decussation* at the lower part of the medulla oblongata. Two, three, or sometimes five nervous filaments are seen to spring from the lateral substance of the medulla; those on the right side cross over the anterior fissure, and go upwards to form the left of those little eminences which anatomists have called, from their shape, corpora pyramidalia; those again, on the left, cross over, and form the right corpus pyramidale. This decussation has been long talked of by anatomists: Aretæus mentions it (see *Aretæus de Caus. et Sig. Morb.* lib. i. cap. 7, p. 34, edit. Lugd. Bat.); and Vicq. D'Azyr has given a representation of it in his plates, which proves that he had never really seen its true place. Subsequent anatomists sometimes affirmed, and sometimes denied its existence; Dr. Spurzheim has the unquestionable merit of having settled the contest for ever, by reviving the knowledge of this important part, and rendering it the object of ocular demonstration.

The pyramidal bodies thus begun, rise upwards in longitudinal medullary fibres, which pass into the pons Varolii. Here they are covered by a superincumbent stratum of transverse fibres, from the lateral lobes of the cerebellum. This stratum being cut, and gently removed by the handle of the scalpel *wetted*, the longitudinal white fibres of the pyramids are distinctly seen passing, without any break or interruption, through the pons; here they are intermixed with brown or cineritious matter, from which an accession of white medullary fibres seems to be derived, for they soon emerge from behind the pons so considerably increased as to form the anterior and external portion of the crura cerebri. Here, in their progress upwards, they are continually receiving new junctions of white fibres emanating apparently from the brown matter in the centre of the crura. But it is at the corpora striata that these bundles receive their greatest additions. In fact, these corpora are considered by Dr. S. merely great *ganglia*, or accumulations of brown matter, from which an infinite number of fine white fibres are produced, diverging in all directions. These being superadded to the anterior portion of the crura cerebri, which we have already seen greatly reinforced and increased, form a multiplied series of radiating fibres (like the shoots of an umbelliferous plant, so to speak), which form at last some of the convolutions of the brain. In this way, the pyramidal bodies being in their progress upwards successively increased, and at last completely developed, go to form nearly the whole of the anterior lobes, and part of the middle lobes of the brain. Hence, from injuries done to these parts of the brain, paralysis will take place on the opposite side of the body, on account of the decussation of the pyramidal bodies, (the primitive bundles to which these parts of the brain are superadded), at the lower part of the medulla oblongata. This is a pathological remark of great importance, and we request our readers to bear it in mind.

It now behoves us to say, before going farther, how the posterior part of the crura cerebri is formed: for this purpose, we must return to the medulla oblongata, and remark the small eminence called corpora olivaria, which lay outwards and laterally of, but in contact with, the corpora pyramidalia. These olive-shaped bodies then do *not decussate* at their lower extremity, as we have seen the pyramids do. On the contrary, the one on the right side is in communication with the right side of the spinal marrow, and the left is in similar communication with all the parts left of the mesial fissure of the medulla oblongata and spinalis. Thus formed, the corpora olivaria ascend through the pons: here, like the corpora pyramidalia, their fibres become imbedded in brown matter, from which they go forth considerably increased, so as to form the posterior and interior portion of the crura cerebri. In their farther progress upwards, they receive constant additions of fibres from the brown matter within. But their greatest reinforcement takes place at what anatomists have called thalami nervorum opticorum, which do not, as is generally believed, give origin to the optic nerves, but are, like the corpora striata, merely great ganglia or masses of brown matter, from which innumerable white fibres issue to join the bundles from below. These united, diverge in every direction, the anterior fasciculi passing into the part of the corpora striata, situated in the lateral ventricles, from which they receive still farther increase. Ultimately, all these fibres, thus successively added to each other, are expanded into the convolutions, and form the posterior lobes of the brain, and the other cerebral parts toward the upper and middle aspect of the head. Hence, it should follow, that injuries done to these parts of the brain, should not produce paralysis on the opposite side of the body ; because the corpora olivaria, of which these lobes seem to be the gradual perfecting and completion, do *not* decussate in the medulla oblongata, like the pyramids. .

The pathology of injuries of the brain is a subject hitherto very insufficiently investigated, and imperfectly understood : indeed, we suspect that the great contrariety of effects said by authors to have resulted from such injuries, cannot be accounted for in any other way than by very freely disbelieving the accuracy of the observers or reporters thereof. We would, therefore, like to see cases more minute in all their details, and bearing specially on this physiological question, instead of the loose and apparently casual notices of them to be found in medical or chirurgical writings. By the way, we may refer for the truth of these remarks, to a most extraordinary paper on the functions of the brain and nervous system, known to come from the pen of the writer of the work now before us, and which is inserted in "the Edinburgh Review" for February 1815, at p. 439. We repeat that it is a most extraordinary paper, and appeal to every enlightened member of our profession who may have read it, for the truth of our remark. That the functions of *organic* or *automatic* life may go on merely by the aid of the nerves, without the brain, we will readily admit; but, after the destruction of that organ, as well as of the spinal marrow, that the functions of *animal* life, such as sensation, volition, and thought, can be performed without detriment, is to us a supposition perfectly incredible and monstrous: and we cannot but lament that any case should have been recorded, *apparently* countenancing a thing so manifestly impossible, so ineffably absurd.

Dr. Spurzheim has, therefore, we think, conferred an evident benefit on science, in calling the attention of physiologists and physicians afresh to the subject of these injuries. From paralysis, as their consequence, being occasionally observed to be propagated on the opposite side of the body to that of the cerebral lesion, a decussation was formerly, indeed, inferred and argued about; but from similar effects, in other cases, being observed to take place on the same side of the body with that of the injury, all was again thrown into doubt and conjecture. The gentleman just mentioned has, however, brought forward a *rule,* founded on the basis of anatomy, by which we may know when paralysis is to take place on the same or on the opposite side. The inferior, outer, and anterior convolutions of the anterior and middle lobes, being in direct communication with, and as it were engrafted on the corpora pyramidalia, will, on account of the decussation, inflict, when injured, paralysis on the opposite side of the body. Whereas all the other convolutions of the brain-proper, being in direct communication with the corpora olivaria, and also the gyri of the cerebellum, whose mass may be considered the gradual completion of the corpora restiformia, (little eminences on the lateral parts of the medulla oblongata) being in communication with the spinal marrow of the same side, whenever injured, will cause paralysis on the same side with that of the injury.

Dr. S. is also of opinion, that though the brain is unquestionably the *medium* of the mind, yet the loss or destruction of any portion of one hemisphere, is not necessarily followed by a loss of faculties; because the brain, as was formerly observed, is a *double* organ, and the sound hemisphere will, in many cases, continue to perform the function of both, in the same manner as we continue to see with the remaining eye, after one of our visual organs has become extinct. He holds, therefore, that none of the cases recorded of loss or destruction of brain, from injury or suppuration, are conclusive against his idea of its nature, and of its being essential to the functions of animal life, unless it could be shewn that all the intellectual powers, together with voluntary motion, continued unimpaired, even after the loss or destruction of *corresponding portions* of *both* hemispheres.

The correctness of the rule here brought forward by Dr. Spurzheim, and of the opinion that the brain is a double organ, must be left to the determination of subsequent experience; and we request our readers to have an eye to this, in the professional progress of their future life. Meanwhile, from the mere *reason* of the thing, we are disposed to think favourably of it, and to believe that Dr. S. has struck into a path which will speedily lead to something certain; for it appears to us, that the point cannot be long doubtful, seeing it admits of the direct evidence of the senses, and of an appeal to actual examples of paralysis from cerebral injury, which are so often occurring. Among the cases recorded by authors, there is such a want of precision as to the *exact* site of the injury, that no determinate judgment can be deduced from them: and as for the instances recorded of the *loss* of brain, they have so much of the marvellous about them, that they ought to be received with the greatest caution. Considerable quantities of brain are, indeed, said to have come away in the process of suppuration; but from our own observation of such cases, we can assure our readers that the matter so discharged, has, in every instance we have met with, been in reality *not* cerebral substance, but a morbid *secretion* from the diseased surface of the brain underneath, which, we suspect, has too often been mistaken for deliquesced portions of that organ.

(To be continued.)

