# Errata

**Published:** 1817-08

**Authors:** 


### ERRATA.

P. 55, l. 12 from top, *for* reviews, *read* views.

55, l. 7 from bottom, *for* descriptions, *r.* discussions.

56, l. 10, *for* Reiley, *r.* Reil.

57, l. 3 from bottom, *for* vol. iii. *r.* vol. ii.

